# Real-World Efficacy and Safety of an 8-Week Glecaprevir/Pibrentasvir Regimen in Children and Adolescents with Chronic Hepatitis C—Results of a Multicenter EpiTer-2 Study

**DOI:** 10.3390/jcm12216949

**Published:** 2023-11-06

**Authors:** Malgorzata Pawlowska, Krystyna Dobrowolska, Justyna Moppert, Maria Pokorska-Śpiewak, Mariola Purzynska, Magdalena Marczynska, Dorota Zarebska-Michaluk, Robert Flisiak

**Affiliations:** 1 Department of Infectious Diseases and Hepatology, Faculty of Medicine, Collegium Medicum Bydgoszcz, Nicolaus Copernicus University, 87-100 Torun, Poland; mpawlowska@cm.umk.pl; 2 Department of Paediatrics, Infectious Diseases and Hepatology, Voivodeship Infectious Observation Hospital in Bydgoszcz, 85-030 Bydgoszcz, Poland; 3 Collegium Medicum, Jan Kochanowski University, 25-317 Kielce, Poland; krystyna.dobrowolska98@gmail.com; 4 Department of Children’s Infectious Diseases, Medical University of Warsaw, 01-201 Warsaw, Poland; maria.pokorska-spiewak@wum.edu.pl (M.P.-Ś.); magdalena.marczynska@wum.edu.pl (M.M.); 5 Department of Pediatric Infectious Diseases, Regional Hospital of Infectious Diseases in Warsaw, 01-201 Warsaw, Poland; 6 Pomeranian Centre of Department of Infectious Diseases and Observation for Children, Smoluchowskiego 18, 80-214 Gdansk, Poland; mariola@purzynscy.pl; 7 Department of Infectious Diseases and Allergology, Jan Kochanowski University, 25-317 Kielce, Poland; dorota1010@tlen.pl; 8 Department of Infectious Diseases and Hepatology, Medical University of Białystok, 15-540 Bialystok, Poland; robert.flisiak1@gmail.com

**Keywords:** HCV infection, pangenotypic DAA therapy, children

## Abstract

The aim of the study was to analyze the effectiveness and safety of anti-HCV treatment based on a pangenotypic direct-acting antiviral (DAA) regimen with glecaprevir/pibrentasvir (GLE/PIB) in children. The multi-center study was conducted in HCV-infected children who were treated in the period from November 2022 to January 2023. The analysis included 23 pediatric patients with a mean (SD) age of 9.61 (3.68) years. The cohort included 13 girls and 10 boys. The most common HCV genotypes were GT1b (*n* = 9, 39.1%), GT1a (*n* = 6, 26.1%) and GT3 (*n* = 5, 21.7%). The SVR was assessed at 12 weeks after the end of treatment and was 100% for both girls and boys. The conducted study showed a very good tolerance of the treatment in the entire analyzed group and confirmed a very high efficacy and safety for 8-week treatment with GLE/PIB in children over three years of age. It seems that our study is the first on the real-world use of an 8-week GLE/PIB pangenotypic therapy in a group of children aged 3–12 years and the first in Europe for adolescents aged 12–17.

## 1. Introduction

Hepatitis C virus (HCV) infection has remained a major public health concern for more than 30 years since its discovery in 1989. Since the introduction of the HCV Elimination Strategy by 2030 by the World Health Organization (WHO) in 2016, its implementation has focused mainly on the detection and treatment of adults whose clinical consequences of this infection lead to liver cirrhosis, liver failure, primary liver cancer—hepatocellular carcinoma (HCC) and death. Compared to adults, little attention has been paid to the diagnosis and treatment of HCV in children and adolescents so far [1,2]. An analysis of the practice of HCV testing and treatment in children in WHO member states published in 2021 showed that most countries (71/122, 58%) lack specific recommendations for the testing and treatment of HCV in children, and only 24/51 countries (mainly in Europe) had specific recommendations for the testing and treatment of HCV in children. Hence, there is an urgent need to update the guidelines for the testing and treatment of HCV infection in children [3].

Achieving the elimination of HCV infections worldwide requires taking into account the pediatric population.

According to Polaris Observatory data, approximately 56.8 million HCV infections were registered worldwide at the beginning of 2020 [4]. The number of HCV-infected children worldwide is estimated at 3.26 million (2.07–3.90) [2]. A meta-analysis of fifty-eight studies showed that the overall prevalence of HCV in children was 0.87%, ranging from 0.34% in Europe to 3.02% in Africa; it did not differ by gender and was higher in children over 10 years of age (0.97%) compared to children under 10 years of age (0.75%, *p* < 0.001) [5].

A retrospective analysis of 1049 patients infected with HCV in childhood indicated that 32% of them developed chronic liver disease over a 30-year period, regardless of the route of infection, while vertically infected patients developed liver cirrhosis at a younger age. In the analyzed group, the incidence of HCC was 5%, liver transplantation was 4%, and death occurred in 3%. A total of 663 patients were treated (55% with interferon/pegylated interferon and 40% with direct-acting antivirals). Sustained viral response (SVR) was achieved in 406 patients (75%) [6]. Early treatment prevents clinical complications and reduces the reservoir of infection.

According to the current WHO recommendations, early treatment of HCV infections in children ensures cure before the development of clinical consequences, both in the context of hepatic and extrahepatic manifestations, prevents the stigmatization of HCV-infected children, and also prevents the transmission of infection, which is particularly important in teenagers. WHO pays special attention to the standards of high-quality services for young people, taking into account the satisfaction of psychological and medical needs, convenience and accessibility of services, the vulnerability of adolescents and their special groups: homeless, living on the streets, orphans, MSM, sexually abused or trafficked adolescents [7].

The high effectiveness and safety of direct-acting antiviral (DAA) therapies in age groups over 3 years, small numbers of severe adverse events (SAEs) and premature termination of therapy in clinical trials, and the approval of DAA therapy for children by the Food and Drug Administration (FDA) and European Medical Agency (EMA) in 2020–2021 are the bases for recommending the use of this treatment in routine clinical practice [7]. Cost-effectiveness analyses comparing treatment at age 6 vs. delaying treatment until age 18 have shown that delaying treatment until age 18 increases the lifetime risk of late hepatic complications, and early treatment in children is cost-effective [8]. According to the recommendations of the Polish Group of HCV Experts, it is recommended to treat HCV infections in all untreated children and after failure of previous therapy. Histological assessment of the liver is not a mandatory eligibility criterion for treatment.

Therapy should be provided in centers with experience in treating children with chronic hepatitis C. The basic therapeutic scheme is interferon-free therapies, which can be used in children over 3 years of age, regardless of the severity of liver disease [9]. Pangenotypic therapies, sofosbuvir/velpatasvir (SOF/VEL) and glecaprevir/pibrentasvir (GLE/PIB), are recommended for the treatment of HCV-infected children without cirrhosis or with compensated cirrhosis (Child-Pugh A). In adolescents over 12 years of age, these therapies are used in adult doses of SOF/VEL—400 mg/100 mg and GLE/PIB—300 mg/120 mg, while in children aged 3–11 the doses depend on the patient’s body weight [9].

The DORA clinical trials (Parts I and II) demonstrated very high efficacy of 8-week therapy with GLE/PIB in adolescents [10] and children aged 3–12 years [11]. The therapy was safe and well tolerated. Data on the efficacy and safety of DAA therapies in children in clinical practice are limited. Previous studies in young children have focused on other, non-pangenotypic therapeutic options, such as SOF/LDV.

Our paper presents the first experience in Europe of using an 8-week GLE/PIB pangenotypic therapy to treat HCV infections in children in routine clinical practice.

## 2. Materials and Methods

### 2.1. Study Population

The analyzed data were collected from pediatric patients diagnosed with hepatitis C who were treated at specialized centers located in Gdańsk, Bydgoszcz and Warsaw in Poland from November 2022 to January 2023. Children were referred to local Hepatology Clinics functioning within Infectious Diseases Departments. Of the 25 patients treated from 2022–2023 with GLE/PIB regimen, 23 were included in the study. Two patients were excluded due to being 18 years old at the time of treatment and therefore considered to be adults. Treatment was based on a DAA regimen with glecepravir/pibrentesvir (GLE/PIB) administered for 8 weeks. The dosage and formulation of the treatment were determined by the physician, based on product characteristics and the Polish Group of Experts (PGE) recommendations [12]. Adolescents aged 12 years and older and children weighing at least 45 kg received treatment in tablet form at the recommended dose of 3 tablets once a day. Children aged 3 to 12 years and weighing 13 to 45 kg received GLE/PIB in granular formula at a dose based on body weight [13].

### 2.2. Data Collection

The data were collected retrospectively using a nationwide database of treated HCV-infected patients of the multi-center EpiTer-2 project which is a research program supported by PTEiLChZ (the Polish Society of Epidemiologists and Infectious Disease Physicians). A general analysis was performed with an additional one depending on the form of drug administration (tablets vs. granules). Parameters studied included demographic and clinical characteristics such as gender, age, body mass index (BMI), HCV genotype, comorbidities, hepatitis B virus (HBV) and human immunodeficiency virus (HIV) co-infection, treatment characteristics and safety. All of the information was collected and then uploaded to the database by the leading physician. Genotyping was performed using reverse hybridization assays, while the assessment of HCV RNA was performed by real-time polymerase chain reaction assays. The presence of kidney disease was assessed using estimated glomerular filtration rate (eGFR) and the patient’s medical history. HBV co-infection was ruled out based on the constellation of serological markers: hepatitis B virus surface antigen (HBsAg), anti-hepatitis B core antibodies (anti-HBc), hepatitis B virus surface antigen antibodies (anti-HBs), and if necessary, molecular markers: HBV desoxyribonucleic acid (HBV DNA). HIV co-infection was assessed using the 4th generation HIV test which detects the presence of the p24 antigens and/or antibodies against HIV (anti-HIV).

### 2.3. Assessment of Liver Disease Severity

The degree of hepatic fibrosis was non-invasively assessed using transient elastography (TE) and defined as F0–F4 according to the METAVIR scale, the values in kilopascals included in the European Association for the Study of the Liver (EASL) recommendations were used for conversion [14]. Advanced liver fibrosis was defined as F3 and cirrhosis as F4. A history of liver transplantation and HCC was assessed based on medical history and imaging studies (ultrasonography). Decompensation of liver function in the past was assessed based on medical history, while at the start of the therapy it was assessed based on the Child-Pugh score.

### 2.4. HCV Treatment Regimen

The effectiveness endpoint was sustained virological response (SVR). It was defined as undetectable HCV ribonucleic acid (RNA) at least 12 weeks after the end of treatment (EOT). Patients with detectable viral load 12 weeks after EOT measurement were considered virologically unresponsive. Per-protocol analysis was carried out by excluding patients lost-to-follow-up who were considered non-virologic failures due to lack of HCV RNA evaluation.

### 2.5. Assessment of Safety

Safety data were collected during treatment and for 12 weeks after EOT. The information collected included modification or discontinuation of therapy, occurrence of adverse events (AEs), severe AEs, and deaths.

### 2.6. Ethics

The patients were not exposed to any experimental interventions and were treated with registered drugs in accordance with PGE recommendations [12]. Patient data was collected and analyzed in accordance with the applicable principles of personal data protection, therefore informed consent was not required. Due to the observational, non-interventional nature of the study with the use of marketed drugs, according to the local law in force at the time of the study (Pharmaceutical Law of 6 September 2001, Article 37al), the approval of an ethics committee was not required.

### 2.7. Statistical Analysis

Continuous data were presented as mean, standard deviations (SD), median and interquartile ranges, whereas categorical data were expressed by number and percentage. All patients who initiated the treatment were included in the intent-to-treat (ITT) analysis, while only those who completed 12 weeks of post-treatment follow-up with an HCV RNA evaluation were appraised in the per-protocol (PP) analysis.

## 3. Results

### 3.1. Patients’ Baseline Characteristics and Laboratory Parameters

The analysis included 23 pediatric patients with a mean (SD) age of 9.61 (3.68) years, with the youngest and oldest aged 3 and 17, respectively. There was a slight preponderance of girls (56.5% vs. 43.5%). The most common HCV genotypes were GT1b (*n* = 9, 39.1%), GT1a (*n* = 6, 26.1%) and GT3 (*n* = 5, 21.7%). Liver stiffness was assessed in 12 patients, of whom 11 (47.8%) had fibrosis grade F0. One patient (4.3%) had fibrosis F1; none had cirrhosis (F4). None of the patients reported a liver transplant or hepatocellular carcinoma. One patient (4.3%) was diagnosed with HBV co-infection, but antiviral treatment did not lead to reactivation of the infection. One patient (4.3%) was reported to have coexisting kidney disease, none of the patients were taking any concomitant medications (Table 1).

Additional analysis based on formulation showed a slight preponderance of girls in the granule group (61.5% vs. 38.5%). The genotype distribution was different between the groups, with only one patient (10%) presenting GT1a and taking tablets as compared to five (38.5%) patients treated with granules. The number of patients with other genotypes was comparable between the two groups. Fibrosis F1 was found in one patient treated with tablets. HBV co-infection and kidney disease were found in two separate patients, both of whom were taking antiviral tablets (Table 2).

The median (IQR) HCV RNA measured at baseline was 645,000 IU/mL (229,324–1,865,000), alanine transaminase (ALT) activity 46 IU/L (37.5–58.5) for the entire group (Table 3).

The median HCV RNA in the granules group was 735,000 IU/mL (107,000–2,030,000) and was higher than in the tablet group: 521,500 (353,000–1,437,500). ALT and bilirubin levels were slightly higher in the tablet group (47.5 vs. 43 IU/L and 0.51 vs. 0.43 mg/dL, respectively), while platelet counts were lower (242 vs. 367 ×1000/µL). The median creatinine was elevated in patients treated with tablets compared to the group receiving the drug in the form of granules (Table 4).

### 3.2. Treatment Effectiveness and Safety

All analyzed patients completed the course of treatment, none of them required treament modification. The SVR assessed at 12 weeks after the end of treatment was 92.3% in the intent-to-treat (ITT) analysis for girls and 100% for boys (Table 5).

**Figure 1:**
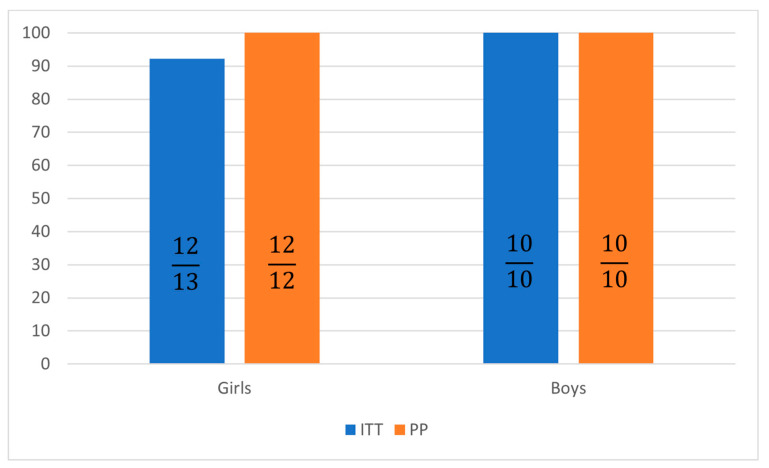
Sustained virological response 12 weeks after treatment in intent-to-treat (ITT) and per protocol (PP) analysis.

**Figure 2:**
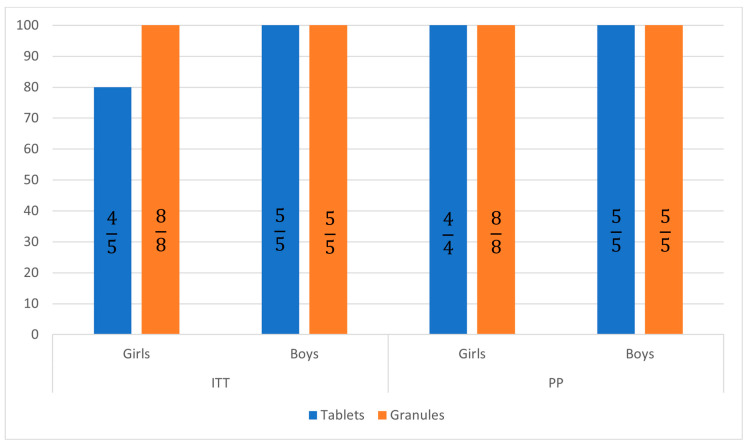
Sustained virological response 12 weeks after treatment in patients treated with tablets and granules in intent-to-treat (ITT) and per protocol (PP) analysis.

Per-protocol (PP) analysis showed a loss of one patient to follow-up, resulting in increasing SVR rates at 12 weeks to 100% in girls treated with GLE/PIB tablets (Figure 1 and Figure 2).

Adverse events were rare and presented as nausea (*n* = 1, 4.3%) and fatigue (*n* = 1, 4.3%). None of these were associated with modification or discontinuation of therapy (Table 3). Both occurred in the tablet-treated group (Table 6).

## 4. Discussion

Pangenotypic therapy with GLE/PIB used successfully in adults with chronic hepatitis C virus (HCV) infection is characterized by very high efficiency, safety and tolerability. So far, 26 articles have been published as part of the EpiTer-2 program, concerning only adult patients. A list of these studies is available at—http://www.pteilchz.org.pl/informacje/epiter-2/ accessed on 6 September 2023.

The first results of using this therapy in children were presented in the DORA clinical trial—a phase 2/3 non-randomized, open-label study evaluating the pharmacokinetics (PK), safety and efficacy of GLE/PIB in children and adolescents with chronic HCV infection. The first part of this study evaluated the efficacy and safety of GLE/PIB at adult-level doses (300 mg/120 mg) once daily for 8 to 16 weeks in 47 adolescents aged 12–17 years infected with genotypes 1, 2, 3 or 4 HCV. All patients (100%) achieved SVR12. No failures or virologic relapses were observed during treatment. No serious adverse events (SAEs) occurred, no adverse events (AEs) led to treatment discontinuation. This pangenotypic regimen was 100% effective in the adolescent population after only 8 weeks of treatment [10]. In part two of the DORA study, the pharmacokinetics, efficacy and safety of the pediatric formulation of GLE/PIB (granules) was evaluated in 80 children aged 3 to <12 years. Children were divided into three age cohorts (9 to <12 years, 6 to <9 years and 3 to <6 years) and received a weight-determined GLE/PIB dose for 8, 12 or 16 weeks. Pediatric doses of GLE/PIB based on pharmacokinetic studies were: 250 mg/100 mg for children ≥30 to <45 kg, 200 mg/80 mg for children ≥20 to <30 kg and 150 mg/60 mg for children weighing 12 to <20 kg. SVR12 was achieved by 77/80 (96%) of patients. One child relapsed at week 4 post-treatment; no child experienced virologic failure. Two patients prematurely completed the study. The most common AEs (occurring in ≥10% of participants) were headache (14%), vomiting (14%), and diarrhea (10%). There were no serious adverse drug-related events. Pediatric GLE/PIB was highly effective and well tolerated in children aged 3 to <12 years with chronic HCV infection [11]. In both parts of the study, GLE/PIB pharmacokinetic exposure was comparable to that in adults.

The DORA clinical trial was the basis for the registration of GLE/PIB therapy by both the FDA and EMA. GLE/PIB therapy is recommended for the treatment of children infected with HCV from 2020 by EASL, from 2021 by AASLD, from 2022 by WHO. In Poland, pangenotypic therapies in the treatment of HCV infections in children are recommended by the Polish Group of HCV Experts from 2020 [12].

Data on the efficacy and safety of GLE/PIB in routine clinical practice in children are limited. Individual reports included real-world experience with GLE/PIB treatment in adolescents with chronic HCV. These data are limited to three Japanese publications, including one original paper and two case reports. The original study evaluated the real-world efficacy and safety of 8–12 weeks of GLE/PIB treatment in Japanese adolescents with chronic HCV. Twenty-five patients infected with HCV genotypes 1b, 2a, 2b and 2b/1b, including 15 girls and 10 boys, were enrolled in the multicenter study. The median age was 13 years (range 12–17). A total of 12/25 (96%) adolescents achieved SVR. Most adverse events were mild, no SAEs were observed [15]. The next two articles describe the high efficacy of an 8-week GLE/PIB therapy in three adolescents. SVR12 was obtained in all three cases and the therapy was very well tolerated. No SAE or early discontinuation of therapy were observed. After DAA therapy, the emotional functioning of patients and their mothers improved [16,17].

Mothers of children with chronic hepatitis C virus (HCV) infection feel anxious about the health of their children. A multi-center survey conducted at six centers in Japan found that successful treatment significantly reduced mothers’ concerns about their children’s HCV infection, indicating that childhood treatment is beneficial for mothers’ psychological burden [18].

Our research is the first study in Europe describing the treatment of HCV-infected children with an 8-week GLE/PIB pangenotypic therapy in routine clinical practice (real-world). It unequivocally confirms the very high (100%) effectiveness, safety and good tolerance of the currently shortest antiviral therapy in the treatment of HCV in adolescents and children, including in the group of the youngest children. It also confirms the validity of minimal monitoring, which is of great importance in the case of the pediatric population.

The loss of one patient from the observation, a teenager taking the drug in the form of tablets, highlights the problem of this age group as the most difficult in pediatrics in the context of adherence to therapy. This particular group is also highlighted by the WHO in the context of the implementation of the HCV elimination strategy, pointing to the legitimacy of promoting teen-friendly procedures [7]. The conducted analysis, taking into account the form of administration of the drug granules/tablets, indirectly reflecting age, indicated a higher median HCV viral load and lower mean values of ALT activity in younger children receiving GLE/PIB in the form of granules. The 100% effectiveness of the treatment obtained in this group of children (mean age 7 years) measured by SVR12 and the persistence of undetectable HCV viral load for 6 months after the end of treatment determines, apart from high safety and good tolerance—no AEs—the validity of using this, currently the shortest, therapeutic regimen against HCV in the youngest children. This is particularly important in the context of treatment of vertically acquired HCV infections, which, apart from teenage infections by taking psychoactive substances or risky sexual behavior, is the most common route of transmission of HCV infections in children. Early treatment of children ensuring the eradication of HCV is the prevention of its chronic consequences in the form of cirrhosis and hepatocellular carcinoma. In the study by Modin et al., patients with perinatal exposure developed cirrhosis at a younger age than the rest of the risk groups [6].

The study revealed a higher percentage of younger children infected with HCV genotype 1a compared to teenagers, which requires further observation.

Undoubtedly, the limitation of our study is the low number of patients. However, this is comparable to the only study published to date assessing the real-world efficacy and safety of GLE/PIB in Japanese adolescents. The results of our research on adolescents are consistent with Mizouki’s observations both in terms of the effectiveness of the therapy measured by obtaining SVR12 (90% vs. 96%, respectively) as well as the safety and tolerance of the therapy [15].

Our study seems to be the first study on the real-world use of an 8-week GLE/PIB therapy in a group of children aged 3–12 years, and the first in Europe for adolescents aged 12–17 years.

## 5. Conclusions

The results of our study conducted in routine clinical practice (real-world) confirm previous observations based on clinical trials regarding the high efficacy, safety and good tolerability of the shortest available therapy against HCV infection in the form of an 8-week treatment with GLE/PIB in pediatric patients over 3 years of age. Obtained results implicates continuation of research, which, however, due to the limited population, will be long-term.

## Figures and Tables

**Table 1:** Characteristics of HCV-infected pediatric patients treated with glecaprevir/pibrentasvir for 8 weeks.

Parameters	Values *n* = 23
Age, mean (SD) (years), (min–max)	9.61 (3.68), (3–17)
Gender, *n* (%) (female/male)	13/10 (56.5/43.5)
BMI, mean (SD)	18.64 (4.37)
Genotypes, *n* (%)	
1	15 (65.2)
1a	6 (26.1)
1b	9 (39.1)
2	0 (0)
3	5 (21.7)
4	3 (13)
Fibrosis/stiffness, *n* (%)	
F0	11 (47.8)
F1	1 (4.3)
F2	0
F3	0
F4	0
Unknown	11 (47.8)
HCC history, *n* (%)	0
Liver transplantation history, *n* (%)	0
HIV coinfection, *n* (%)	0
HBV coinfection, *n* (%)	1 (4.3)
Any comorbidity, *n* (%)	1 (4.3)
Renal diseases	1 (4.3)
Concomitant medications, *n* (%)	0

Abbreviations: BMI, body mass index; HBV, hepatitis B virus; HCC, hepatocellular carcinoma; HIV, human immunodeficiency virus; SD, standard deviation.

**Table 2:** Characteristics of HCV-infected pediatric patients treated with glecaprevir/pibrentasvir administered in the form of tablets and granules.

Parameters	Tablets *n* = 10	Granules *n* = 13
Age, mean (SD) (years), (min–max)	13.20 (1.72), (11–17)	7 (2.03), (3–10)
Gender, *n* (%) (female/male)	5/5 (50/50)	8/5 (61.5/38.5)
BMI, mean (SD)	22.51 (3.59)	15.65 (1.86)
Genotypes, *n* (%)		
1	6 (60.0)	9 (69.2)
1a	1 (10.0)	5 (38.5)
1b	5 (50.0)	4 (30.8)
2	0 (0)	0 (0)
3	3 (30.0)	2 (15.4)
4	1 (10.0)	2 (15.4)
Fibrosis/stiffness, *n* (%)		
F0	5 (50)	6 (46.2)
F1	1 (10)	0 (0)
F2	0 (0)	0 (0)
F3	0 (0)	0 (0)
F4	0 (0)	0 (0)
Unknown	4 (40)	7 (53.8)
HCC history, *n* (%)	0 (0)	0 (0)
Liver transplantation history, *n* (%)	0 (0)	0 (0)
HIV coinfection, *n* (%)	0 (0)	0 (0)
HBV coinfection, *n* (%)	1 (10)	0 (0)
Any comorbidity, *n* (%)		
Kidney diseases	1 (10)	0
Concomitant medications, *n* (%)	0	0

Abbreviations: BMI, body mass index; HBV, hepatitis B virus; HCC, hepatocellular carcinoma; HIV, human immunodeficiency virus; SD, standard deviation.

**Table 3:** Baseline laboratory parameters of HCV-infected pediatric patients treated with glecaprevir/pibrentasvir for 8 weeks.

Parameters	Values *n* = 23
HCV RNA, median (IQR) [IU/L]	645,000 (229,324–1,865,000)
ALT, median (IQR) [IU/L]	46 (37.5–58.5)
Albumin, median (IQR) [g/dL]	4.63 (4.46–4.87)
Biblirubin, median (IQR) [mg/dL]	0.46 (0.37–0.56)
Platelets ×1000/µL, median (IQR)	323 (260–378.5)
Creatinine, median (IQR) [mg/dL]	0.46 (0.4–0.57)
Hemoglobin, median (IQR) [g/dL]	13.6 (12.75–13.9)
INR, median (IQR)	1.08 (1.05–1.12)

Abbreviations: ALT, alanine transaminase; HCV, hepatitis C virus; INR, international normalized ratio; RNA, ribonucleic acid.

**Table 4:** Baseline laboratory parameters of HCV-infected pediatric patients treated with glecaprevir/pibrentasvir for 8 weeks.

Parameters	Tablets *n* = 10	Granules *n* = 13
HCV RNA, median (IQR) [IU/mL]	521,500 (353,000–1,437,500)	735,000 (107,000–2,030,000)
ALT, median (IQR) [IU/L]	47.5 (42.75–64.25)	43 (33–49)
Albumin, median (IQR) [g/dL]	4.70 (4.63–5.1)	4.57 (4.30–4.72)
Biblirubin, median (IQR) [mg/dL]	0.51 (0.45–0.68)	0.43 (0.34–0.50)
Platelets ×1000/µL, median (IQR)	242 (197–313)	367 (305–416)
Creatinine, median (IQR) [mg/dL]	0.62 (0.54–0.77)	0.42 (0.39–0.45)
Hemoglobin, median (IQR) [g/dL]	13.80 (13.60–13.90)	13 (12.40–13.8)
INR, median (IQR)	1.10 (1.06–1.11)	1.06 (1.04–1.12)

Abbreviations: ALT, alanine transaminase; HCV, hepatitis C virus; INR, international normalized ratio; RNA, ribonucleic acid.

**Table 5:** Treatment characteristics and the safety of antiviral therapy in HCV-infected pediatric patients treated with glecaprevir/pibrentasvir for 8 weeks.

Parameter	Values
Number of patients, *n*	23
Treatment course, *n* (%)	
According to schedule	23 (100)
Therapy modification	0
Therapy discontinuation	0
Patients with at least one AE, *n* (%)	
Nausea	1 (4.3)
Fatigue	1 (4.3)

Abbreviations: AE, adverse event.

**Table 6:** Treatment characteristics and safety of antiviral therapy in HCV-infected pediatric patients treated with glecaprevir/pibrentasvir for 8 weeks.

Parameter	Tablets	Granules
Number of patients, *n*	10	13
Treatment course, *n* (%)		
According to schedule	10 (100)	13 (100)
Therapy modification	0 (0)	0 (0)
Therapy discontinuation	0 (0)	0 (0)
Patients with at least one AE, *n* (%)		
Nausea	1 (10.0)	0 (0)
Fatigue	1 (10.0)	0 (0)

Abbreviations: AE, adverse event.

## Data Availability

Data supporting the reported results can be provided upon request from the corresponding author.
